# *In vitro* and *in vivo* acute toxicity of a novel citrate-coated magnetite nanoparticle

**DOI:** 10.1371/journal.pone.0277396

**Published:** 2022-11-17

**Authors:** Jose Marcos Vieira Rocha, Valeria Barbosa de Souza, Patricia Costa Panunto, Jacqueline Spacagna Nicolosi, Emanueli do Nascimento da Silva, Solange Cadore, Oscar Moscoso Londono, Diego Muraca, Pablo Tancredi, Marina de Brot, Wilson Nadruz, Ana Lucia Tasca Gois Ruiz, Marcelo Knobel, Andre Almeida Schenka

**Affiliations:** 1 Department of Pharmacology, School of Medical Sciences, University of Campinas (UNICAMP), Campinas, Brazil; 2 Institute of Chemistry, UNICAMP, Campinas, Brazil; 3 Department of Chemistry, Institute of Exact and Biological Sciences, Federal University of Ouro Preto, Ouro Preto, Brazil; 4 Institute of Physics "Gleb Wataghin", UNICAMP, Campinas, Brazil; 5 Laboratory of Amorphous Solids, INTECIN, Faculty of Engineering, University of Buenos Aires–CONICET, Buenos Aires, Argentina; 6 Department of Anatomic Pathology, A. C. Camargo Cancer Center, Campinas, Brazil; 7 Department of Internal Medicine, School of Medical Sciences, UNICAMP, Campinas, Brazil; 8 School of Pharmaceutical Sciences, UNICAMP, Campinas, Brazil; 9 Department of Anatomic Pathology, School of Medical Sciences, UNICAMP, Campinas, Brazil; Hamadan University of Medical Sciences, ISLAMIC REPUBLIC OF IRAN

## Abstract

Magnetic nanoparticles (MNps) have become powerful tools for multiple biomedical applications such as hyperthermia drivers, magnetic resonance imaging (MRI) vectors, as well as drug-delivery systems. However, their toxic effects on human health have not yet been fully elucidated, especially in view of their great diversity of surface modifications and functionalizations. Citrate-coating of MNps often results in increased hydrophilicity, which may positively impact their performance as drug-delivery systems. Nonetheless, the consequences on the intrinsic toxicity of such MNps are unpredictable. Herein, novel magnetite (Fe_3_O_4_) nanoparticles covered with citrate were synthesized and their potential intrinsic acute toxic effects were investigated using *in vitro* and *in vivo* models. The proposed synthetic pathway turned out to be simple, quick, inexpensive, and reproducible. Concerning toxicity risk assessment, these citrate-coated iron oxide nanoparticles (IONps) did not affect the *in vitro* viability of different cell lines (HaCaT and HepG2). Moreover, the *in vivo* acute dose assay (OECD test guideline #425) showed no alterations in clinical parameters, relevant biochemical variables, or morphological aspects of vital organs (such as brain, liver, lung and kidney). Iron concentrations were slightly increased in the liver, as shown by Graphite Furnace Atomic Absorption Spectrometry and Perls Prussian Blue Staining assays, but this finding was considered non-adverse, given the absence of accompanying functional/clinical repercussions. In conclusion, this study reports on the development of a simple, fast and reproducible method to obtain citrate-coated IONps with promising safety features, which may be used as a drug nanodelivery system in the short run. (263 words)

## 1. Introduction

Nanotechnology encompasses innovative tools dedicated to the design, synthesis, and application of materials with at least one dimension at the nanoscale (or one billionth of a meter, usually 0.1-100nm) [1–4]. Because of their size and surface area/mass ratio, nanomaterials display unique chemical, electronic, optical, and magnetic properties, which can be useful for biomedical applications such as drug delivery systems and biological molecular probes [1, 5–6].

Iron oxide nanoparticles (IONps) such as magnetite (Fe_3_O_4_) and maghemite (γ-Fe_2_O_3_) have been widely explored in biomedical applications due to their magnetic behavior, besides their generally assumed low toxicity [7–9]. The physical and chemical properties of magnetic nanoparticles allow them to be applied as imaging contrast probes, hyperthermia inducing agents, magnetic-guided vectors, and drug delivery carriers [10–15], among other potential uses. When magnetic nanoparticles are small enough, they behave as giant paramagnetic materials (i.e., showing lack of hysteresis at room temperature), thus being designated as superparamagnetic iron oxide nanoparticles (SPIONs). SPIONs can be biocompatible particles, and some of them have already been used as contrast medium in magnetic resonance imaging and in cancer therapy [16–19].

The use of SPIONs, such as magnetite particles, as drug vehicles may result in the reduction of drug dosing and, consequently, of toxic side effects [20]. Nonetheless, some studies have shown concern about the intrinsic toxicity of certain nanomaterials, especially in view of their wide biodistribution, persistence in vital metabolic active tissues and wide variety of surface modifications and functionalizations [21–23]. Such surface intentional alterations may result for instance in pharmacokinetic/pharmacodynamic benefits, but at the cost of increased toxicity. For this reason, thorough toxicological evaluation of synthetic nanomaterials is essential and mandatory for many regulatory agencies. Citrate-coating of MNps, in particular, often results in increased hydrophilicity, which may improve their performance as drug-delivery systems. However, the consequences of such surface functionalization on the intrinsic toxicity of the modified MNps are unpredictable.

Despite increasing use of *in vitro* tests (as surrogates to established *in vivo* methods, given pressing ethical and economic concerns), such methods do not allow for a complete toxicopathological assessment of nanomaterials. *In vitro* tests have not provided reliable prediction of systemic toxicity, including adverse phenomena arising from pharmacokinetic/pharmacodynamic interactions of test nanocomposites and their metabolic derivatives (toxicokinetics) [24–27].

Organisation for Economic Co-operation and Development (OECD) test guideline No. 425 [28] has been used in most *in vivo* nanotoxicity publications, thus proving to be an equally effective and ethical instrument to assess the potential acute oral toxicity of such materials. In addition to median lethal dose (LD_50_) estimation, these guidelines also recommend observation of clinicopathological signs of toxicity, thus allowing for the classification of the tested substance according to the Harmonized Global System for Classification of Chemicals [29].

In this context, the present study aimed to describe the synthesis and characterization of novel coated magnetite (Fe_3_O_4_) nanoparticles, as well as to assess their safety profile using *in vitro* (cell viability of HaCaT and HepG2 cell lines) and *in vivo* (oral acute toxicity test in Sprague-Dawley rats) assays. To the best of our knowledge, this is the first study assessing “in vitro” toxicity of citrate-coated magnetite nanoparticles.

## 2. Material and methods

### 2.1. Synthesis of citrate-coated Fe_3_O_4_ nanoparticles (citrate-coated IONps)

The novel IONps were synthesized by the co-precipitation method. A mixture of FeCl_3_·6H_2_O (3.7 mmol) and FeCl_2_·4H_2_O (1.85 mmol) dissolved in H_2_O (25 mL) was added dropwise to a NaOH solution (7.5 g in 125 mL of H_2_O) under argonium atmosphere and vigorous stirring. After 30 min, citric acid (5 g) was added to produce hydrophilic-carboxyl coated Fe_3_O_4_ nanoparticles. The temperature was then raised to 80°C and maintained for 30 minutes. Citrate-coated Fe_3_O_4_ nanoparticles were magnetically decanted and subsequently dispersed in H_2_O to obtain a stable ferrofluid (pH = 7).

### 2.2. Citrate-coated IONps characterization

#### 2.2.1. X-ray diffraction analysis (XRD)

XRD measurements were performed on a Phillips PW1820/1710 diffractometer using Cu Kα radiation with 1.54 Å wavelength, in a 2θ range from 20° to 70° and a scan step of 0.01°/s on the nanoparticles loaded cellulose acetate films.

#### 2.2.2. Transmission electron microscopy (TEM)

The novel IONps were dispersed in water and sonicated for 15 minutes. Samples for TEM observation were then prepared by drying a drop of this suspension during 24 hours at room temperature on a Ted Pella ultrathin copper film on a holey carbon grid. TEM images were obtained at the Brazilian National Nanotechnology Laboratory (LNNano) using an electron microscope (model JEM 2010, at 200 kV, with a spot size of 3nm). Using TEM images captured from more than three hundred random nanoparticles, a histogram was built and properly fitted with a lognormal function for size analysis purposes.

#### 2.2.3. Magnetic field dependence of magnetization at room temperature

Field-dependent magnetization curve was recorded at room temperature using a vibrating sample magnetometer (VSM, Lakeshore 7407), with a magnetic field between -2 and 2 Tesla. For such magnetic measurements, samples were centrifuged and subsequently dried. The obtained powder was weighed and placed into the VSM sample holder.

### 2.3. *In vitro* evaluation

#### 2.3.1. Cell culture

HaCaT cell line (spontaneously transformed keratinocytes from histologically normal skin) was kindly provided by Dr. Ana Lucia T.G. Ruiz (University of Campinas, UNICAMP). HepG2 cell line (hepatocellular carcinoma cells) was obtained by Laboratory of Dr. Gabriel Forato Anhê at University of Campinas. The cell lines were grown in RPMI 1640 (HaCaT) or DMEM (HepG2) media supplemented with 5% fetal bovine serum (FBS 5%) and 1% penicillin: streptomycin mixture (1000 U mL^-1^:1000 μgmL^-1^). These cell lines were incubated at 37°C in a humidified atmosphere of 5% CO_2_. When cells attained 70–80% of confluence, they were collected and used in the assays.

#### 2.3.2. Cell viability: The MTT (3-(4,5-dimethylthiazol-2-yl)-2-5 diphenyl tetrazolium bromide) assay

HaCaT (10^4^ cells/well) and HepG2 (1.5 x 10^4^ cell/well) cells were exposed to citrate-coated IONps (at concentrations ranging from 0.0001–100 μg/ml), for 72 hours. Doxorubicin hydrochloride (Pfizer, Brazil) treated cells (at concentrations ranging from 0.0001–100 μg/ml) and untreated cells were used as positive and negative controls, respectively. At the end of treatment protocols, the cells were assessed for viability using MTT assays.

We followed the MTT protocol as described by Mosmann [30]. Briefly, for each cell line, the supernatant was removed, the cells were washed with phosphate-buffered saline (PBS, pH 7.0, 100μl/well) and then they were incubated with MTT solution (Sigma Aldrich, 0.5 mg/mL in PBS, 100μl/well) for 4 hours at 37°C in a humidified atmosphere of 5% CO_2_. After this, the 96-well plates were centrifuged, and the supernatant removed. The insoluble formazan crystals were dissolved in Isopropyl alcohol (150μl/well). The absorbance was read in a SpectraMax 340PC 384 microplate reader (Molecular Device, 1311 Orleans Drive Sunnyvale, CA 94089) at 570 nm.

Cell viability results were normalized using the absorbance of untreated cells (which was considered as 100% viability). The half maximal inhibitory concentration (IC50) was calculated by plotting cell viability versus citrate-coated IONps concentration (on a log10 scale) followed by sigmoidal fitting (Prism Graphpad software, version 5.0). IC_50_ values were reported as mean ± standard deviation (SD) of three independent experiments, each performed in quintuplicate.

### 2.4. *In vivo* evaluation

#### 2.4.1. Animals

Female Sprague-Dawley rats (6 to 8 weeks, weighing between 200 ± 60 g) were obtained from the Multidisciplinary Center for Biological Research (CEMIB)/UNICAMP. The experimental protocol was approved by the Ethical Committee for Animal Experimentation of the University of Campinas (Unicamp) under the protocol number CEUA n° 3838–1. Animals were randomly housed in groups (5/cage) within cages with stainless steel covers to allow acclimatization. The environmental conditions were 12 h day/night cycle, temperature 22 ± 2°C, relative humidity 55 ± 10%, standard diet and water *ad libitum*.

#### 2.4.2. Acute oral toxicity test

The single dose protocol used to evaluate citric acid-coated IONps followed the Organisation for Economic Co-operation and Development (OECD), guideline 425 [27]. Eight Sprague-Dawley rats were randomly distributed into 2 groups named (1) experimental group/EG (animals receiving a single dose of citrate-coated IONps, at 2000 mg/kg, diluted in 2mL of filtered water, by oral gavage; n = 4), and (2) control group/CG (animals receiving 2mL of filtered water, also by oral gavage; n = 4). First, one animal of each group was treated and observed for clinical signs of toxicity. After 48 h without any toxic sign, the other four animals of each group were treated. After administration, all animals were continuously observed for clinical signs of toxicity and possible mortality during the first four hours and every day for 14 days. At the first, seventh and fourteenth days, animal body weight was measured. After 14 days of observation, all animals were anesthetized with isoflurane (Cristália, Brazil) and euthanized by cervical dislocation. The brain, liver, spleen, heart, kidney, muscle, eyes, intestine, stomach, pancreas, sexual organs, skin and bladder of each animal were collected, weighed and examined macroscopically for lesions and/or abnormalities. Relative organ weight was calculated by dividing each animal’s organ weight by their body weight. Furthermore, part of these organs was collected, fixed in 10% neutral buffered formalin and processed for histological analysis, while a small fresh sample of each organ was maintained at -80°C for spectrometric assessment.

#### 2.4.3. Biochemical analysis

At days 0 (baseline) and 14 of treatment, a blood sample (500 μL) was draw from each experimental animal by retro-orbital plexus puncture using heparinized capillaries which were then poured into microcentrifuge tubes. After centrifugation (2500 rpm, for 5 minutes, at 4°C), plasma samples were submitted to biochemical analysis, using commercially available kits (all from Bioclin/Quibasa, Minas Gerais, Brazil), including the quantitation of kinetic creatinine (KC, cat. #K067), creatine kinase MB (CK-MB, cat.# K069), total creatine kinase (total CK, cat.# K010), urea (U, cat.# K056), lactic dehydrogenase (LDH cat.# K014), aspartate aminotransferase (ALT, cat.# K048), alanine aminotransferase (ALT, cat.# K049), and alkaline phosphatase (ALP, cat.# K021). All samples were read on a Beckman Coulter DU 800 spectrometer (Beckman Instruments, California, USA).

#### 2.4.4. Histopathological evaluation

After 48h of fixation in 10% neutral buffered formalin, the tissues were processed, embedded in paraffin blocks and cut into 5-μm sections using a microtome. All tissue sections were submitted to H&E staining, while spleen and liver sections were also stained with Perls Prussian Blue to detect and quantify iron deposits in these organs [31]. Images from these slides were obtained through whole slide scanning using an automated system (Aperio AT2 slide scanner, Leica Biosystems) with fixed parameters for magnification (40x) and resolution (0.252 microns/pixels). Random hotspot images of iron deposits (as highlight by the Perls staining) were selected and save as TIFF files, out using the Aperio ImageScope software.

#### 2.4.5. Perls’ Prussian blue staining protocol

We used Mallory´s modification of Perls’ stain. First, we deparaffinized and hydrated the sections with distilled water. Then, we washed the slides (containing spleen and liver sections from control and treated animals) with working solution, and we heated the slides in the microwave oven for 30 seconds. Subsequently, the slides were rinsed in distilled water. Next, they were incubated with nuclear-fast red for 5 minutes. Finally, the slides were washed in tap water, dehydrated, and coverslip with mounting medium. Blue areas/dots (iron deposits) on selected images (5 mediuam power fields/animal) were quantified using the Image J software (NIH, USA).

#### 2.4.6. Determination of Fe concentration in rat tissues by Graphite Furnace Atomic Absorption Spectrometry

In order to determine Fe concentration in the tissues, deionized water (9.5 mL) was added to each organ sample (0.1–1.0 g, depending on the organ size) and allowed to stand for 5 minutes. Subsequently, tetramethylammonium hydroxide (0.5 mL, TMAH 25%) was added and the mixture stood, at least, for 1h in an ultrasound cleaning (for complete solubilization). The analysis was performed by graphite furnace atomic absorption spectrometry (GF AAS model A Analyst 600, Perkin-Elmer, Norwalk, USA) at the 248.3 nm, and some posterior dilution was made with deionized water if necessary. The analytical calibration curve was obtained at concentrations of 10–80 μgL^-1^ using aqueous metallic element standards and Mg (NO_3_)_2_ as a chemical modifier (15 μg/analysis). The pyrolysis and atomization temperatures were 1400°C and 2100°C, respectively. The same procedure was carried out with two certified reference material (NIST 1577b and NIST 8414) to determine the method’s accuracy. All readings were performed in triplicate. The instrumental conditions for Fe quantification were optimized by evaluating the recoveries of certified reference materials. The results showed that suitable recoveries were obtained (103 ± 5% and 103 ± 3% for the 1577b and 8414 materials, respectively), as well as adequate relative deviations (below than 10%). Limits of Detection (LoD) and Quantification (LoQ) were calculated, and the values were 2.5 and 8.2 μg L-1, respectively. All assessments were made in triplicate.

### 2.5. Statistical analysis

Differences between control and citrate-coated IONp treatment groups were determined using Student’s *t*-test for body and relative organ weights, and Fe concentration analysis (both by Graphite Furnace Atomic Absorption Spectrometry and by Prussian blue staining). The paired *t*-test was used with biochemical variables. In all analysis, the significance level was established at *p* <0.05.

## 3. Results

### 3.1. Nanoparticles synthesis and characterization

**Fig 1:**
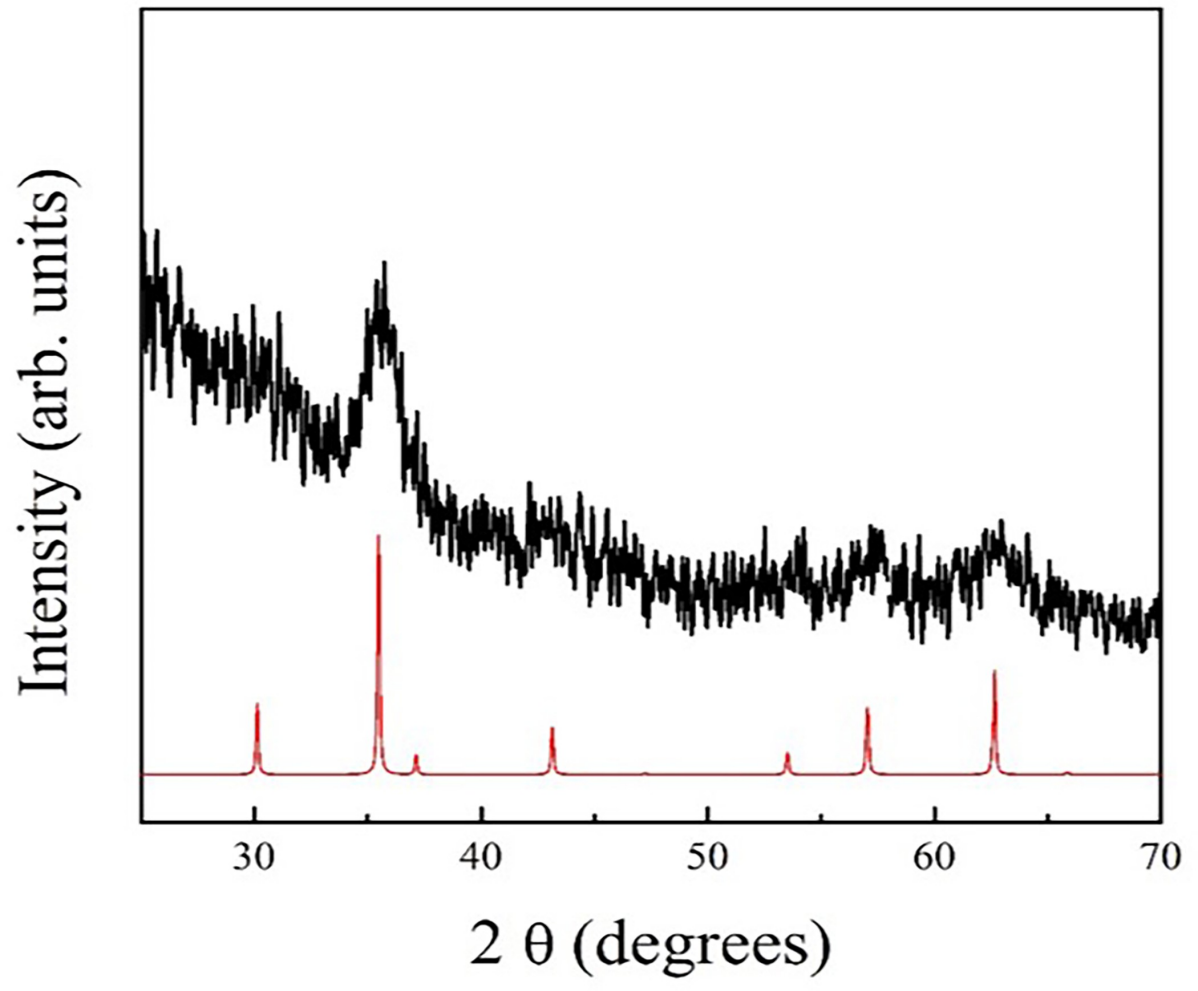
X-Ray diffraction pattern of citrate-coated iron oxide nanoparticles. Black peaks represent citrate-coated IONps and red peaks, ICSD-data base.

**Fig 2:**
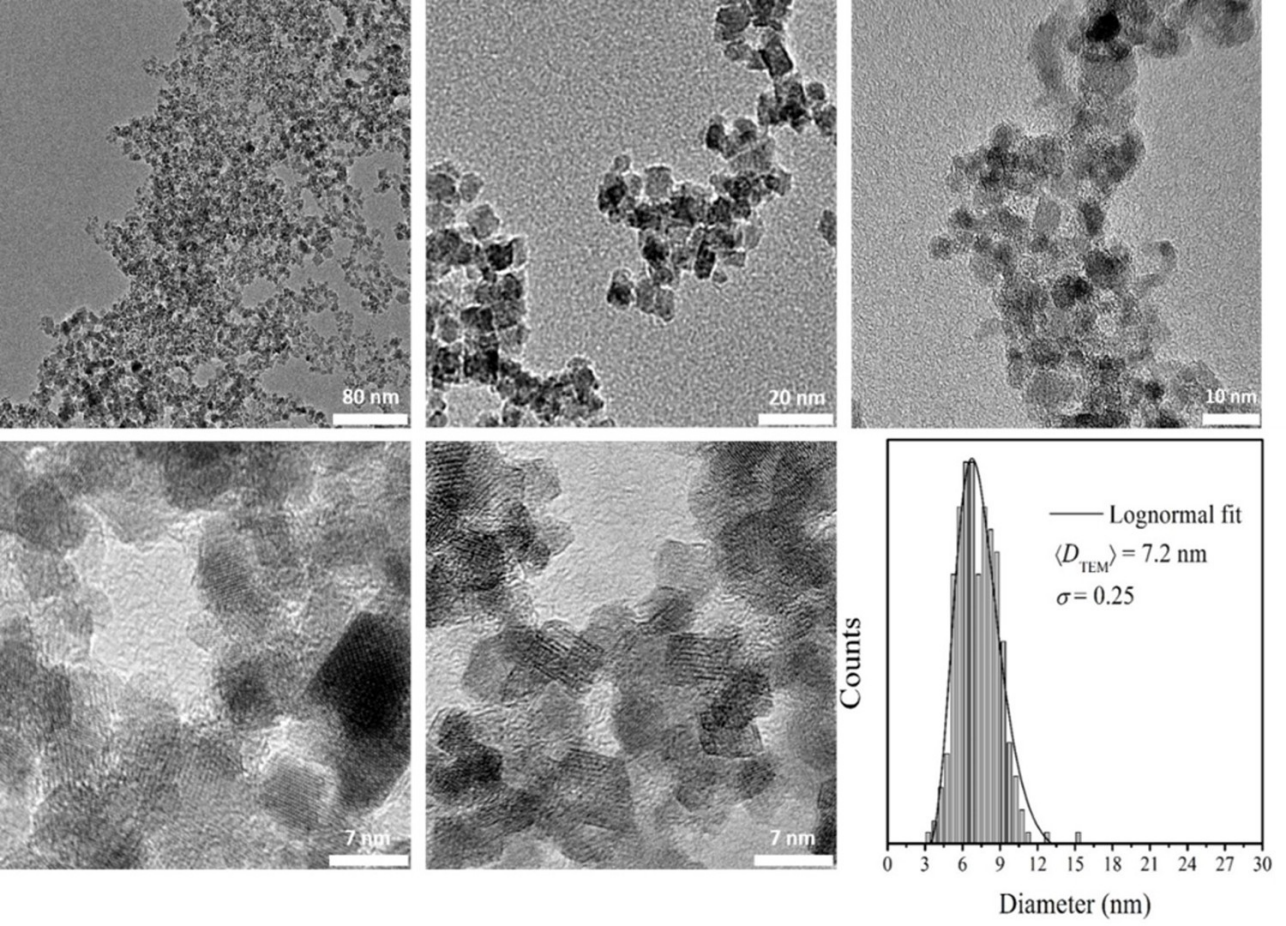
TEM images and size distribution of the magnetite nanoparticle (IONps).

The diffractogram for citrate-coated IONps is characteristic of small size particles and matches the Fe3O4 or Ɣ-Fe2O3 (maghemite) crystalline phase, confirming the formation of the fcc spinel-inverse iron oxide phase (Fig 1). In addition, the most intense reflection peaks were associated to the magnetite phase at 2θ = 35.4° (311), 56.9° (511) and 62.5° (440) (Fig 2). TEM images analysis indicated that the IONp is composed by aggregated and non-aggregated nanoparticles with three predominant morphologies (spherical, hexagonal and square). The nanoparticle aggregation may have occurred during the dehydrating steps of the tissue processing for microscopic analysis. Histogram analysis indicates that the mean diameter of the nanoparticles is 7.2 ± 0.25 nm. The measurements of interplanar distances (using Bragg’s law) are consistent with the lattice spacings of Fe3O4 or Ɣ-Fe2O3 crystals. Furthermore, both the size and morphology of the nanoparticles are consistent with the employed synthesis procedure.

**Fig 3:**
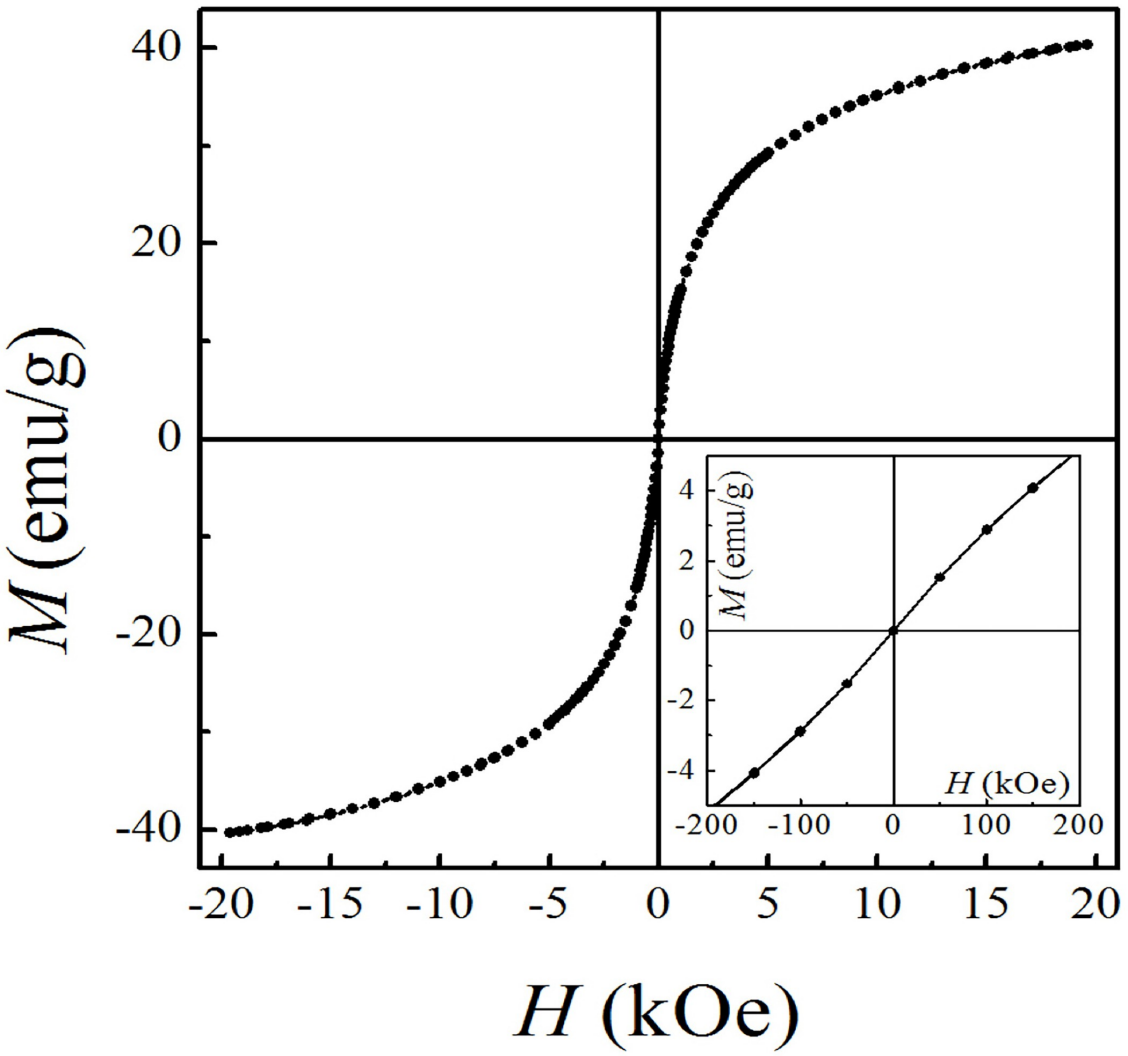
Magnetization as a function of applied field measured at room temperature (IONps). Low field behavior is shown in the inset.

Fig 3 shows magnetization as a function of the applied field at room temperature. The inset zooms the low applied field region. The saturation magnetization (obtained from Langevin formalism) was 42 emu/g. As a first approximation, one can say that the experimental curves seem to be typical of systems with single domain nanoparticles, showing a null coercive field, which is characteristic of a system in thermodynamic equilibrium. This fact indicates that the nanoparticles did not agglomerate due to magnetic interactions, which is fundamental for the use in biomedical applications.

### 3.2. Effect of citrate-coated IONps on HaCaT and HepG2 cells viability

**Fig 4:**
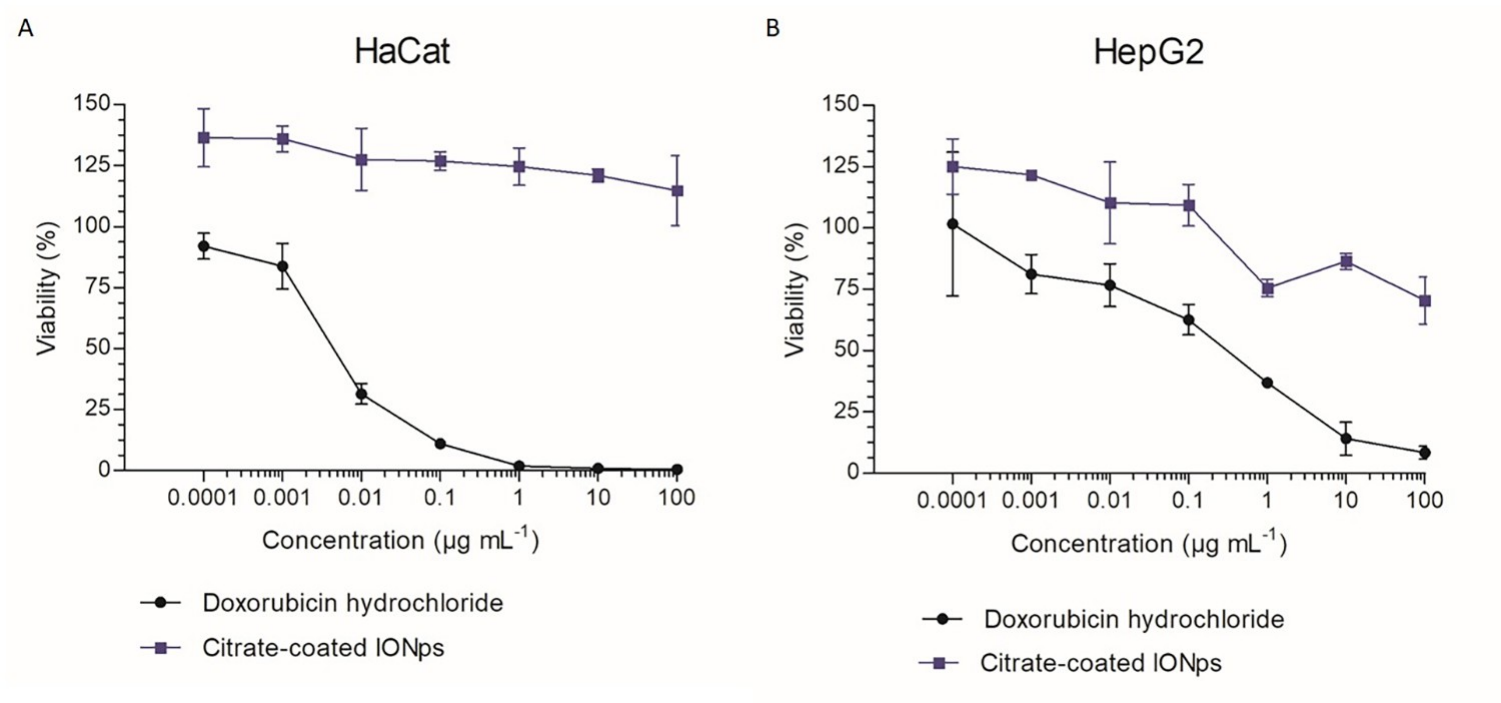
Cell viability after 72-h-exposition to citrate-coated IONps and doxorubicin hydrochloride. The human HepG2 (hepatocellular carcinoma, **A**) and HaCaT (spontaneously transformed keratinocytes, **B**) cell lines were treated with different concentrations (100 to 0.1 ug/mL) of citrate-coated IONps, and the viability was measured 72h later with MTT assay. The blue line represents the nanoparticle and the black line corresponds to doxorubicin hydrochloride (positive control).

Fig 4 shows the growth curve of HepG2 (hepatocelular carcinoma) and HaCaT (spontaneously transformed keratinocytes) cell lines after treatment with citrate-coated IONps using MTT assays. Citrate-coated IONps demonstrated IC_**50**_ values greater than 100 μg/mL for both cell lines tested, indicating lack of significant toxicity. In contrast, doxorubicin hydrochloride (positive control) reduced the viability of HepG2 (IC_**50**_ = 0.452 ± 0.312 μg/mL) and HaCaT (IC_**50**_ = 0.004 ± 0.001 μg/mL) cell lines after 72-h exposition, at very low doses, as expected.

### 3.3. *In vivo* effect of citrate-coated IONps

**Fig 5:**
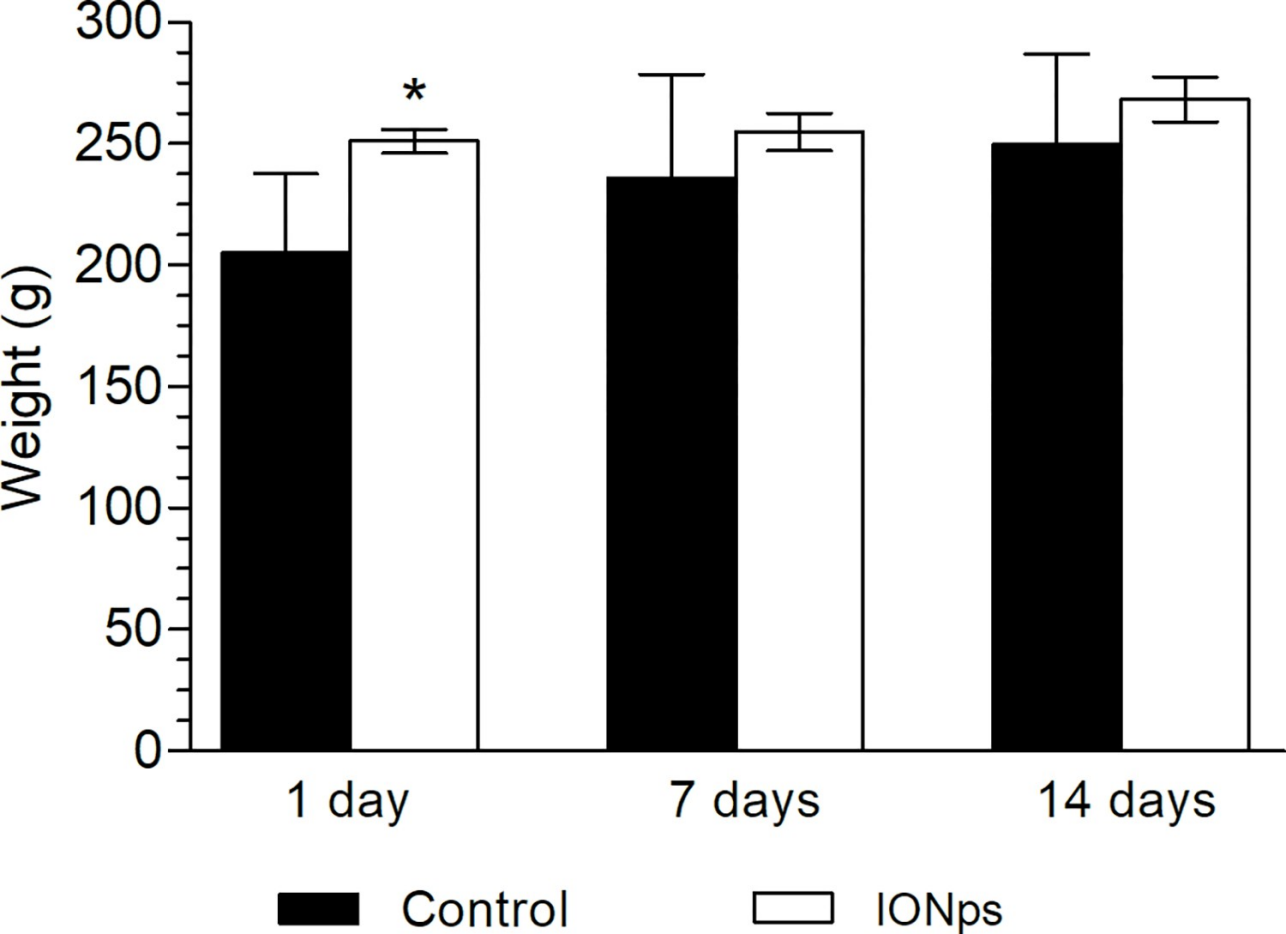
Average body weight of female Sprague-Dawley rats during the acute oral toxicity experiment. Results were expressed as mean ± standard deviation; Groups: control (filtered water, 2 ml/animal, n = 4), citrate-coated IONps (diluted in filtered water, 2000 mg/kg, 2 ml/animal, n = 4). Statistical analysis by Student’s T test (* *p <* 0.05) relating to the control group.

We investigated the toxicity profile of citrate-coated IONps in Sprague-Dawley rats using OECD guideline 425 (OECD, 2008). In this experiment, all animals survived after one dose of citrate-coated IONps (2000 mg/kg), and none of them showed any clinical signs of toxicity, such as convulsion, nausea, vomiting or diarrhea, breathing difficulties or irritation, during the 14 days of the protocol. The rats maintained their regular food and water intake which can be confirmed by the body weight gain curve. All animals showed a similar weight gain profile during the 14 days following the treatment (Fig 5). Further, no significant change in relative organ weight (including brain, liver, spleen, kidney and lung), were observed when comparing control animals with citrate-coated IONp-treated individuals (Table 1).

**Table 1:** Relative weight (% relative to final body weight) of heart, kidney, spleen, liver, lung and brain at day 14 of the acute oral toxicity experiment with citrate-coated IONps.

Groups	Heart	Kidney	Spleen	Liver	Lung	Brain
Control	0.329±0.0002	0.449±0.0003	0.242±0.0002	5.952±0.0038	0.546±0.0007	0.740±0.0004
Citrate-coated IONps	0.322±0.000*	0.398±0.0003	0.333±0.0006	4.058±0.0056	0.725±0.0011	0.748±0.0002

Results were expressed as mean ± standard deviation; Animals: female Sprague-Dawley rats (6 to 8 weeks, weighing between 200 ± 60 g); Groups: control (potable water, 2 ml/animal, n = 4), citrate-coated IONps (diluted in potable water, 2000 mg/kg, 2 ml/animal, n = 4). Statistical analysis by Student’s T test (* *p <* 0.05) relating to the control group.

**Fig 6:**
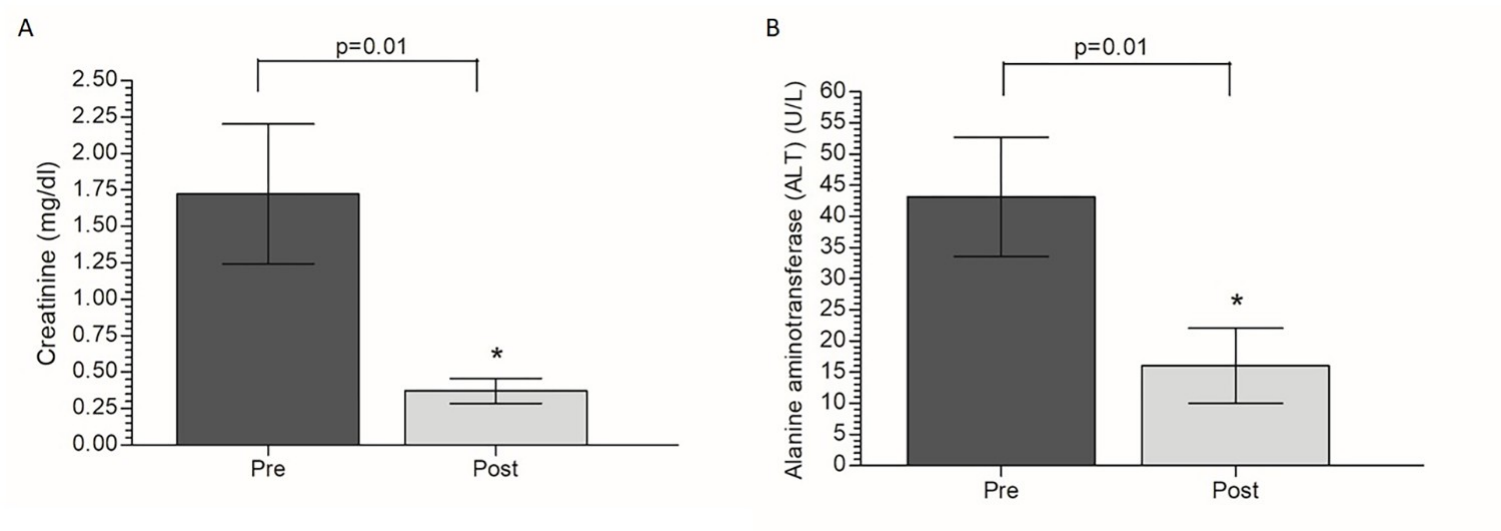
Evaluation of (A) Creatinine and (B) ALT plasma levels at the 14-day of the acute oral toxicity experiment of citrate-coated IONps. Results were expressed as mean ± standard deviation; Animals: female Sprague-Dawley rats (6 to 8 weeks, weighing between 200 ± 60 g); Groups: Pre (animals before citrate-coated IONps treatment, n = 4), Post (animals at the 14-day after citrate-coated IONps treatment, n = 4). Statistical analysis by Student’s T test (* *p <* 0.05) relating to the basal evaluation.

Considering biochemical parameters, the plasma samples from pre (day 0) and post (day 14) treatment with citrate-coated IONps were investigated for possible effects on hepatic (AST, ALT, alkaline phosphatase), cardiac and non-cardiac muscle (LDH, CK-MB and CK-Total), and renal (creatinine and urea) functions (Table 2). Of notice, statistically significant differences in the mean values of ALT and creatinine were observed between pretreatment and post-treatment groups (Fig 6).

**Table 2:** Biochemical plasma parameters at days 0 (baseline/pre-treatment) and 14 (post-treatment) of the acute oral toxicity protocols using citrate-coated IONps.

	LDH (U/l)	CK-MB (U/l)	CK-total (U/l)	AST (U/l)	ALT (U/l)	Creatinine (mg/dl	ALP (U/ml)	Urea (mg/dl)
Pre	208.82±143	165.41±33	86.24±71	112.75±33	43.16±10	1.72±0.5	24.44±2	38.13±11
Post	108.77±62	160.13±78	27.1±10	97.65±42	16.05±6*	0.37±0.1*	24.53±3	73.62±29

Results were expressed as mean ± standard deviation; Animals: female Sprague-Dawley rats (6 to 8 weeks, weighing between 200 ± 60 g); Groups: Pre (animals before citrate-coated IONps treatment, n = 4), Post (animals at the 14-day after citrate-coated IONps treatment, n = 4). Statistical analysis by Student’s T test (* *p <* 0.05) relating to the basal evaluation.

**Fig 7:**
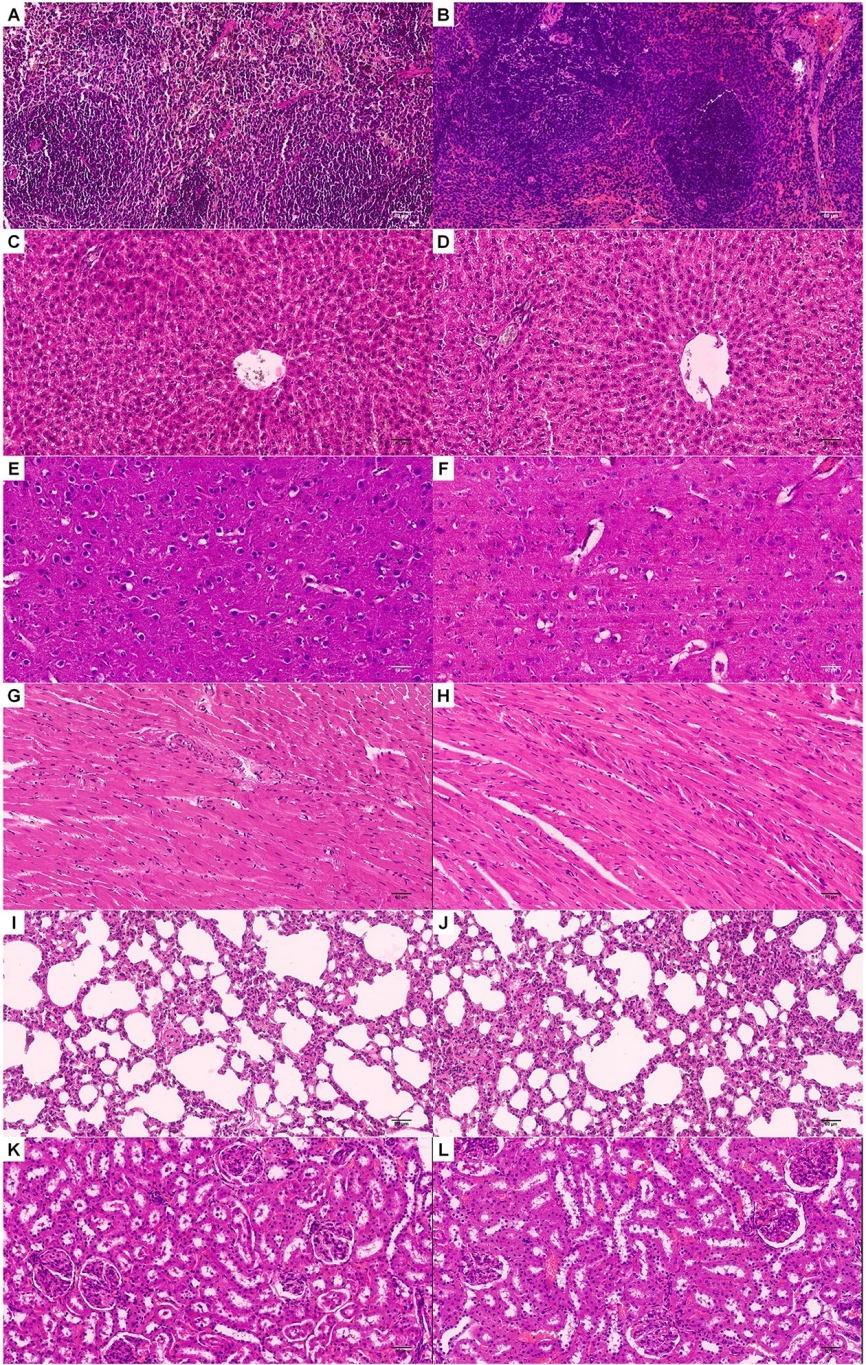
Photomicrograph of spleen section of H&E staining of Sprague-Dawley. **(A, B)** spleen, **(C, D)** liver, **(E, F)** brain, **(G, H)** heart, (I,J) lung and **(K, L)** kidney. Animals: female Sprague-Dawley rats (6 to 8 weeks, weighing between 200 ± 60 g); A, C, E, G, I and K: control group (filtered water, 2 ml/animal, n = 4); B, D, F, H, J and L: citrate-coated IONps group (diluted in filtered water, 2000 mg/kg, 2 ml/animal, n = 4). Scale bar: 50μm, 10x magnification.

**Fig 8:**
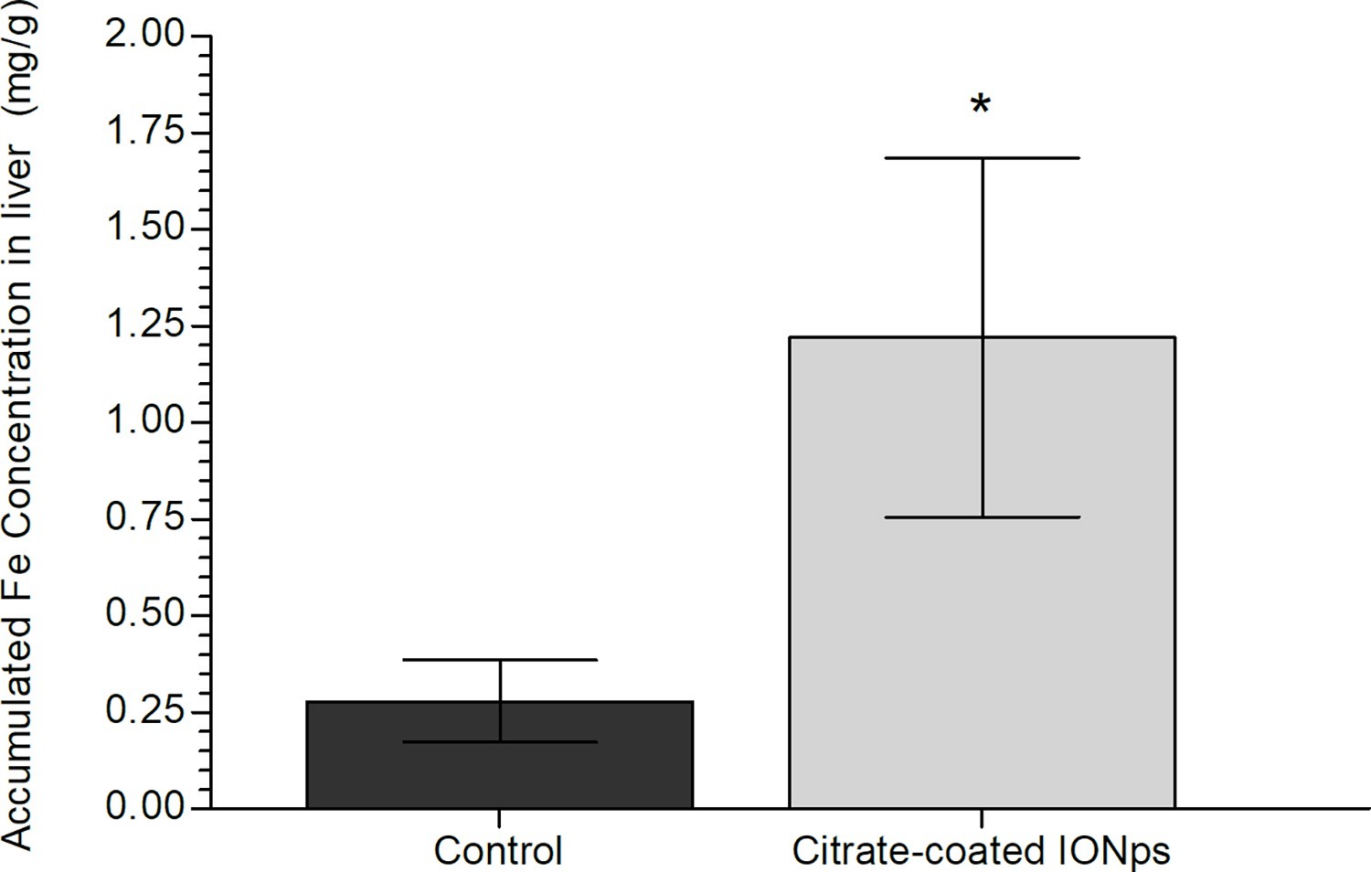
Fe concentration analysis by Graphite Furnace Atomic Spectrometry in liver at 14-day of the acute oral toxicity experiment of the citrate-coated IONps.

**Fig 9:**
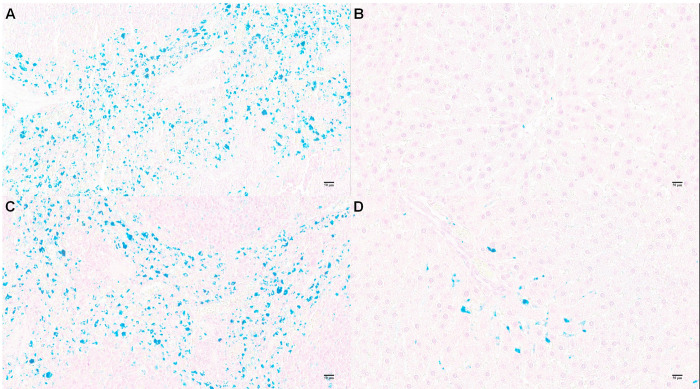
Fe concentration analysis by Prussian blue staining in spleen **(A, C)** and liver **(B, D)** at 14-day of the acute oral toxicity experiment of the citrate-coated IONps. Animals: female Sprague-Dawley rats (6 to 8 weeks, weighing between 200 ± 60 g); A and B: control group (potable water, 2 ml/animal, n = 4); C and D: citrate-coated IONps group (diluted in potable water, 2000 mg/kg, 2 ml/animal, n = 4). Scale bar: 50μm, 20x magnification. Note that Fe stained by Prussian blue in liver (circle and arrow) is lower concentration then spleen.

**Fig 10:**
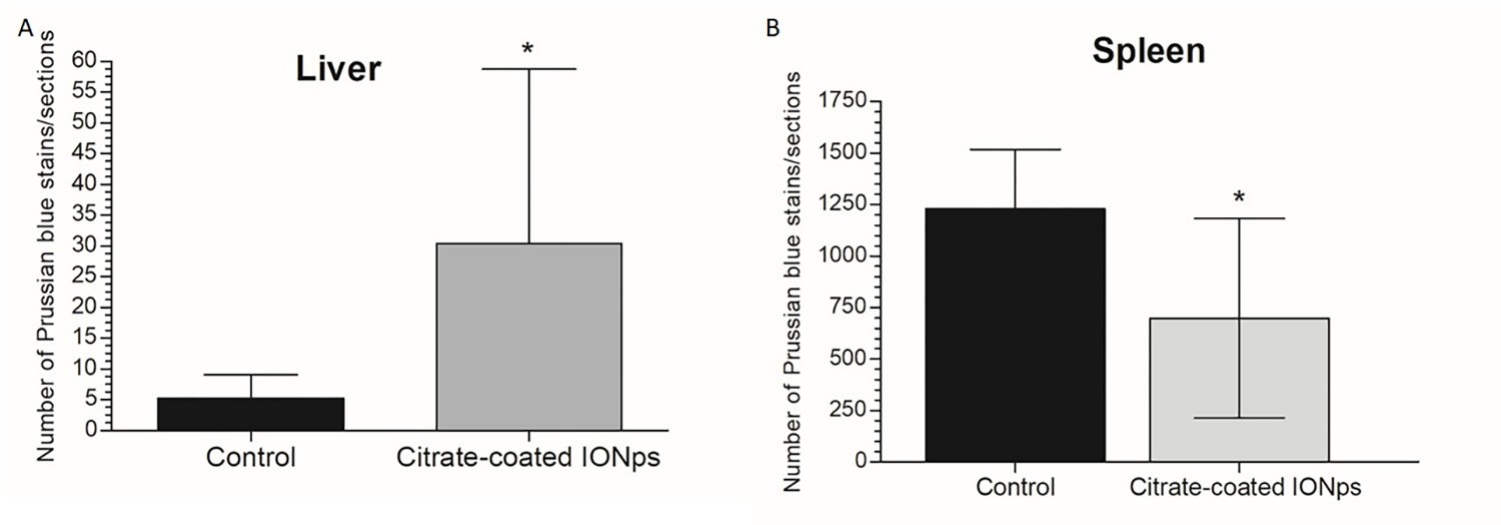
Number of Prussian blue positive profiles after exposure to citrate-coated IONps. The graphs show quantification of the average number of Prussian blue positive staining per section from Sprague-Dawley administered control (vehicle) and treated (citrate-coated IONps). A: Liver; B: Spleen. Statistical analysis by Student’s T test (* *p <* 0.05) relating to the control group.

Histological examination of major organs (brain, heart, liver, lung, kidney, spleen) using H&E stained sections was performed in all experimental and control animals. Comparing citrate-coated IONp-treated animals with those of control group, no remarkable lesions were observed that could be attributed to the treatment (Fig 7). Iron accumulation in liver, heart, lung, brain, kidney and spleen were analyzed by Graphite Furnace Atomic Absorption Spectrometry to determine the total Fe accumulation. Only the liver of citrate-coated IONp-treated animals showed a significant increase in total Fe accumulation in comparison to control group (Table 3, Fig 8). To confirm this result, the histological slides of liver and spleen were further analyzed by the Pearls Prussian Blue Staining (Fig 9). Perls’ stain showed a significant (p < 0.05) increased of iron deposits in the liver samples from rats treated with citrate-coated IONps in comparison to control group, but decreased in the spleen (Fig 10).

**Table 3:** Iron concentration analysis by Graphite Furnace Atomic Spectrometry in vital organs at day 14 of the acute oral toxicity experiment with citrate-coated IONps.

Organs						
Accumulated Fe Concentration (mg/g)	Kidney	Brain	Heart	Lung	Liver	Spleen
Control	0.111±0.033	0.018±0.003	0.163±0.089	0.080±0.046	0.278±0.106	0.250±0.005
Citrate-coated IONps	0.181±0.128	0.016±0.001	0.087±0.020	0.165±0.122	0.997±0.584*	0.324±0.085

Results were expressed as mean ± standard deviation; Animals: female Sprague-Dawley rats (6 to 8 weeks, weighing between 200 ± 60 g); Groups: control (potable water, 2 ml/animal, n = 4), citrate-coated IONps (diluted in potable water, 2000 mg/kg, 2 ml/animal, n = 4). Statistical analysis by Student’s T test (* *p <* 0.05) relating to the control group.

## 4. Discussion

The functionalization of iron oxide magnetic nanoparticles can improve their biomedical applications. Properties such as particle size, particle size distribution, crystallinity and shape are determined by the synthesis method. So, reactions performed in controlled conditions will result in better nanoparticles considering sample homogeneity and synthesis reproducibility [32–34]. Considering the different alternatives, we chose the modified Massart [35] co-precipitation method for the IONps synthesis and citrate as the coating agent.

The co-precipitation method was used due to its simplicity, reproducibility and less hazardous materials and methods, being one of the most promising methods for biomedical applications. In addition, the advantage of small magnetic nanoparticles for biomedical applications is related to their superparamagnetic character, confirmed by the zero-remanence magnetization when the magnetic field is removed. The citrate coating improves the colloidal stability of the IONps by granting a permanent negative charge imposes intense electrostatic repulsion forces between the nanoparticles. This effect reduces any possible magnetic interaction between the nanoparticles, preventing agglomeration and facilitating dispersion in aqueous media. The nanoparticles composition mean size and distribution were confirmed by electron microscopy and X-ray diffraction. Superparamagnetic behavior was observed, as expected for a sample of 7 nm, which is shorter than the critical size for multidomain nanoparticles. So, the absence of coercive field and superparamagnetic like behavior are desired characteristics for nanoparticles suitable for biomedical applications.

In present study, the *in vitro* results indicate that citrate-coated IONps do not affect the viability of normal (keratinocytes, HaCaT) or tumor (hepatocelular carcinoma, HepG2) cell lines up to 100μg/mL. These results are suggestive of an absence of effect on normal tissues proliferation. It is worth mentioning that as an on-target toxicology approach (antiproliferative effect), it was not possible to predict other toxic effects [36].

The *in vivo* experiment on rats was done following the OECD guidelines 425. Animals treated with citrate-coated IONps exhibited neither clinical signs of toxicity, nor alterations on body weight. Alterations in body weight, both in acute and chronic toxicity assays, can be attributed to a direct decrease in food/water intakes and/or to damages into major organs such as the stomach, intestine, liver and kidney [21, 37]. Clinical signs of toxicity can suggest which organs are affected by the test item and if mortality follows these signs, a probable cause of death may be established [24].

Another relevant variable that can be used to assess the occurrence of adverse effects is the relative weight of organs that are primarily affected by toxicants, such as the heart, kidneys, spleen, liver and lungs [38, 39]. In this study, citrate-coated IONps were not associated to a negative impact on the relative organ weight.

Biochemical analysis is very important in the monitoring of clinical symptoms produced by toxicants. Alanine aminotransferase (ALT), aspartate aminotransferase (AST) and alkaline phosphatase (ALP) activities values are indicative of liver function, while creatine kinase (CK) and CK-MB activities are useful in the evaluation of cardiac and non-cardiac muscle functions. In citric acid-coated Fe_3_O_4_ nanoparticle-treated animals, only ALT values were changed, and even so, they were within the limits of normality for the species and lower than baseline values [38].

Corroborating with the absence of hepatic or cardiac damage, there was no significant alteration in LDH plasma level in animals treated with citrate-coated IONps. LDH is a cytoplasmatic enzyme evolved in pyruvate-lactate conversion concomitant to NADH-NAD+ conversion. Increased LDH serum levels are related to pathological conditions of liver, heart, kidney and lung [21, 40, 41]. Regarding renal function, kidney damage or dehydration can be detected through blood analysis of urea, electrolytes and creatinine [21, 42, 43]. Elevated plasma creatinine is a reliable indicator of impaired glomerular filtration or alterations in renal blood flow, but severe tubular dysfunction can also increase plasma creatinine [44, 45]. The citrate-coated IONps promoted a reduction in creatinine levels without changing circulating urea levels. Despite the reduction, the creatinine level agreed with the expected range for the species [40] indicating no toxic effect.

In histopathological analysis, there was no change in vital organs such as brain, spleen, liver, lung and kidney after citrate-coated IONps treatment, further corroborating with the lack of biochemical and clinical evidences for toxicity. In the liver, the hepatocytes were not altered in the treated group despite the increase in iron content in this organ, as evidenced by Graphite Furnace Spectrometry and Perls’ stain. Our results suggested that the iron accumulation did not promote any hepatic injury as also pointed by ALT and AST values.

In conclusion, herein, we described a simple way to synthesize citrate- coated IONps (⦸7.2nm) that could be useful in the future as a nanodelivery system, since no obvious adverse effects were detected. Given the significant (yet non-adverse) concentration of these nanoparticles within the liver, these IONps could be particularly advantageous to the delivery of drugs and diagnostic agents (such contrast media) to this organ.

## Abbreviations

ALT: alanine aminotransferase
ALP: alkaline phosphatase
AST: aspartate aminotransferase
CEMIB: Multidisciplinary Center for Biological Research
CEUA: Ethical Committee for Animal Experimentation
CG: control group
CK: creatine kinase
EG: experimental group
FAPESP: Sao Paulo State Research Support Foundation
FBS: fetal bovine serum
GF AAS: graphite furnace atomic absorption spectrometry
HaCaT: a spontaneously transformed keratinocyte cell line, derived from normal adult human epidermal keratinocytes
HepG2: a neoplastic hepatocyte cell line, derived from hepatocellular carcinoma cells
H&E stain: hematoxylin and eosin stain
IONps: iron oxide nanoparticles
LDH: lactic dehydrogenase
LNNano: Brazilian National Nanotechnology Laboratory
LOD: limits of determination
LOQ: limits of quantification
MNps: magnetic nanoparticles
MRI: magnetic resonance imaging
MTT: 3-(4,5-dimethylthiazol-2-yl)-2-5 diphenyl tetrazolium bromide
OECD: Organisation for Economic Co-operation and Development
PBS: phosphate-buffered saline
SD: standard deviation
SPIONs: superparamagnetic iron oxide nanoparticles
TEM: transmission electron microscopy
TMAH: tetramethylammonium hydroxide
UNICAMP: State University of Campinas
VSM: vibrating sample magnetometer
XRD: X-ray diffraction analysis
